# Is ectopic Cushing’s syndrome underdiagnosed in patients with small cell lung cancer?

**DOI:** 10.3389/fmed.2022.954033

**Published:** 2022-08-30

**Authors:** Marta Piasecka, Martin Larsson, Eleni Papakokkinou, Lena Olsson, Oskar Ragnarsson

**Affiliations:** ^1^Department of Internal Medicine and Clinical Nutrition, Institute of Medicine at Sahlgrenska Academy, University of Gothenburg, Gothenburg, Sweden; ^2^Department of Endocrinology, Sahlgrenska University Hospital, Gothenburg, Sweden; ^3^Department of Respiratory Medicine, Sahlgrenska University Hospital, Gothenburg, Sweden

**Keywords:** ectopic ACTH-production, hypercortisolism, small-cell lung cancer (SCLC), paraneoplastic syndrome, ectopic Cushing’s syndrome

## Abstract

**Introduction:**

Ectopic Cushing’s syndrome (ECS) is an uncommon disorder. Recently, however, a larger proportion of patients with endogenous Cushing’s syndrome (CS) had ECS than has previously been reported.

**Objective:**

The aim of this study was to determine whether ECS is an underdiagnosed disorder in patients with small-cell lung cancer (SCLC).

**Materials and methods:**

Medical records from consecutive patients diagnosed with SCLC at our hospital between 2013 and 2019 were reviewed (*N* = 213; mean age 69.5 ± 9 years; range, 36–89 years). The probability of having ECS was evaluated by review of biochemical and clinical features, including presence of recent onset diabetes mellitus, therapy resistant hypertension and/or spontaneous hypokalaemia.

**Results:**

Of 213 identified patients with SCLC, one (0.5%) patient had confirmed ECS, two (1%) patients had probable ECS, and twenty-three (11%) patients had possibly ECS. Patients with SCLC and possibly or probable ECS exhibited a significantly shorter survival than patients only with SCLC (8 vs. 14 months, respectively).

**Conclusions:**

Our findings indicate that ECS is underdiagnosed in patients with SCLC. Given the serious consequences of untreated ECS, the low detection rate highlights the need to improve endocrine work-up of patients with SCLC who present with biochemical and clinical features associated with ECS. Prospective studies are needed to establish a reliable assessment of the incidence of ECS and to optimise early detection strategies.

## Introduction

Ectopic Cushing’s syndrome (ECS) is an uncommon endocrine disorder caused by autonomous and excessive adrenocorticotropic hormone (ACTH) secretion from a tumour not originating in the pituitary gland (1, 2). The increased ACTH production subsequently causes hypercortisolism, that is often severe and characterised by treatment resistant hypertension, pronounced insulin resistance and hyperglycaemia, severe hypokalaemia and muscle weakness. Furthermore, due to the greatly increased ACTH production, hyperpigmentation of the skin and oral mucosa is also common in patients with ECS. In contrast to patients with Cushing’s syndrome (CS) of pituitary or adrenal origin, hypercortisolism in patients with ECS usually develops rapidly (weeks) and typical features such as central obesity with abdominal striae may be absent (3).

Ectopic Cushing’s syndrome is considered to account for approximately 5–15% of all patients with endogenous CS. Half of these are caused by lung tumours, either bronchial carcinoids or small cell lung cancer (SCLC) (1, 2, 4, 5). Recently, however, we found a higher proportion of ECS among patients with CS than has previously been reported (6). Of 80 patients diagnosed with CS in the Västra Götaland Region between 2002 and 2018, 21 (26%) had ectopic CS, of whom 8 had lung cancer.

In previous reports, between 1 and 6% of patients with SCLC had concomitant ECS (7–10). However, due to atypical presentation, rapid progression and diagnostic difficulties, the prevalence of ECS in patients with SCLC may still be underestimated.

The aim of this study was to evaluate the prevalence of clinical characteristics in patients with SCLC that may be related to ECS. Our main hypothesis is that ECS is an underdiagnosed comorbidity in patients with SCLC.

## Materials and methods

### Design

This was a retrospective study including patients who were diagnosed with SCLC at the Sahlgrenska University Hospital in Sweden between January 1st 2013 and December 31st 2019. Approximately half of all patients diagnosed with lung cancer in the Västra Götaland County, with 1.8 million inhabitants, and all patients with lung cancer diagnosed in north part of the Halland County (around 100 000 inhabitants) are referred to the Department of Respiratory Medicine at the Sahlgrenska University Hospital for evaluation and treatment. In Sweden, diagnostic codes are provided during all hospital visits and registered according to the diagnosis-related group (DRG) registry. To identify patients with SCLC, a search in the DRG-registry at the hospital was performed by using the specific ICD-10 code for SCLC (C34.9B).

### Patients

**FIGURE 1:**
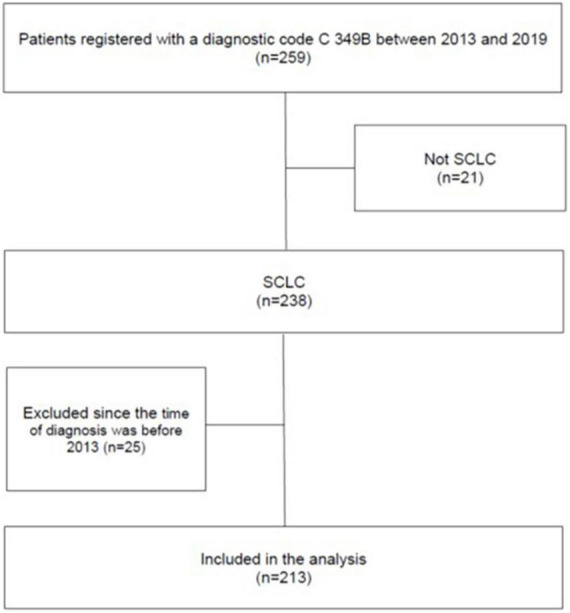
Flow chart showing the selection of patients in the study. The patients were identified by a search in the DRG-registry at the Sahlgrenska University Hospital by using the ICD-10 code for SCLC.

In total, 259 patients who had received a diagnostic code for SCLC during the study period were identified. Of these, 21 patients with histopathologically confirmed lung cancer of other aetiologies than SCLC were excluded. Additionally, 25 patients were excluded since the time of diagnosis was before 2013. Thus, the final cohort consisted of 213 patients with histopathologically confirmed SCLC (Figure 1). None of the patients had previously received a diagnosis of ECS.

### Data collection and identification of patients with ectopic Cushing’s syndrome

The medical records of all patients were reviewed and data on clinical features, biochemical data, imaging and histopathological diagnosis were collected, including data on: (a) new onset therapy-resistant hypertension; (b) new onset diabetes mellitus; (c) clinically significant hypokalaemia (≤3.0 mmol/L); (d) documented hyperpigmentation; and (e) documented presence of Cushingoid features. Therapy-resistant hypertension was defined as high blood pressure despite concurrent use of three different antihypertensive agents (11).

To validate the diagnosis of ECS, the medical records of all patients identified by the DRG registry were reviewed by an experienced endocrinologist. The diagnosis of ECS was considered to be “confirmed,” “probable,” or “possible,” based on the criteria provided in Table 1.

**TABLE 1:** Definitions of confirmed, probable and possible ectopic Cushing’s syndrome used in this study.

Confirmed	Biochemical analyses confirming ectopic CS, including serum cortisol, plasma ACTH, and urinary free cortisol
Probable	Presence of two of the following: (i) new onset therapy resistant hypertension, (ii) new onset diabetes mellitus, (iii) clinically significant hypokalaemia (≤3.0 mmol/L)
Possible	Presence of one of the following: (i) new onset therapy resistant hypertension, (ii) new onset diabetes mellitus, or (iii) clinically significant hypokalaemia (≤3.0 mmol/L)

### Ethics

The study was approved by the Regional Research Ethics Committee in Gothenburg, Sweden (reference number 814–18; approved 26 November 2018) and conducted according to the Declaration of Helsinki.

### Statistics

All data were analysed in IBM SPSS version 25.0.0.0. Categorical data are presented as number of subject (%). Normally distributed variables are presented as mean ± standard deviation (SD) and non-normally distributed variables as median (interquartile range; IQR or range). Kaplan–Meier curves were used to illustrate survival and the Log rank test to estimate the difference between patients with and without ECS.

## Results

In total, 213 patients (128 women, 60%) were diagnosed with SCLC between 2013 and 2019. The mean age at diagnosis was 69.5 ± 9 (range, 36–89) years. Of 213 patients, 14 (7%) had severe hypokalaemia (serum potassium ≤3 mmol/l), 12 (6%) had therapy resistant hypertension and 4 patients (2%) had new-onset diabetes mellitus (Table 2). Cushingoid features were documented in 6 (3%) patients and hyperpigmentation in one. S-Cortisol had been measured in 35 patients, of whom 9 had concentrations at 8 AM >900 nmol/L.

**TABLE 2:** Characteristics of patients without ectopic Cushing’s syndrome (ECS) and with possible ECS.

	All (*N* = 213)	Patients without ECS (*N* = 187)	Possible ECS (1) (*N* = 23)
Age (years), mean ± SD	69.5 ± 9	69 ± 9	70.6 ± 8
Female gender, *N* (%)	128 (60)	110 (58)	17 (71)
Hypertension, *N* (%)	105 (49)	85 (45)	17 (71)
Therapy-resistant hypertension, *N* (%)	12 (5.6)	0	9 (39)
New-onset diabetes mellitus, *n* (%)	4 (1,9)	0	2 (9)
Hypokalaemia (<3 mmol/L K), *N* (%)	14 (7)	0	12 (52)

Based on the criteria presented in Table 1, including presence of therapy-resistant hypertension, new-onset diabetes mellitus, and/or clinically significant hypokalaemia (≤3 mmol/L K), one (0.5%) patient was considered to have confirmed ECS, two (1%) patients had probable ECS, and 23 (11%) patients had possible ECS.

### Confirmed ectopic Cushing’s syndrome

#### Case 1

An active 81-year-old man with a history of smoking and prostate cancer presented with a few months history of muscle weakness, dyspnoea and peripheral oedema. Hyperpigmentation was noticed and documented, but not cushingoid features. At presentation the patient had therapy-resistant hypertension and hypokalaemia (s-potassium 2.9 mmol/L) that required treatment with 6 g potassium chloride daily and spironolactone. He did not have diabetes mellitus. Imaging studies reviled multiple lesions in the lungs and liver metastases. Bronchoscopy was performed and biopsy confirmed SCLC. ECS was suspected and endocrine work-up revealed S-cortisol at 8 AM of 1,850 nmol/L (normal 102–535 nmol/L), urinary free cortisol 3,660 nmol/L (normal 55–215 nmol/L) and p-ACTH 129 pmol/L (normal 2–11 pmol/L). The patient’s condition deteriorated rapidly and 7 days after SCLC was diagnosed he died before any specific treatment was started.

### Probable ectopic Cushing’s syndrome

#### Case 2

A 67-year-old woman with a history of 100 pack-years of smoking, hypertension, primary hyperparathyroidism, osteoporosis and alcohol overconsumption, presented with a pathological radius fracture. Laboratory examination revealed severe hypokalaemia (1.9 mmol/L) and treatment with potassium supplements was started (6 g per day). A few days later, the patient was readmitted due to bowel perforation. Serum potassium was 2.9 mmol/L, despite potassium supplementation. During emergency laparotomy, metastases in the liver and gall bladder were discovered. Histopathological examination revealed SCLC. Chest CT revealed a tumour in the left lung hilum, mediastinal lymph node metastases and pleural effusion.

Due to therapy resistant hypertension and hypokalaemia, screening for secondary hypertension was performed and displayed high s-cortisol (3,370 nmol/L) and normal aldosterone/renin ratio [27 mIU/L (normal 4–65 mIU/L)]. Due to hyperglycaemia treatment with insulin was initiated. No further endocrinological work-up was performed. Fifteen days after SCLC was confirmed, palliative chemotherapy was started. Unfortunately, the patient’s condition deteriorated thereafter, and 3 weeks later she died due to pneumonia with neutropenic fever.

#### Case 3

A 67-year-old man with a history of cardiovascular disease and a recently diagnosed diabetes mellitus presented with dyspnoea on exertion. Clinical examination was consistent with heart failure that was treated with diuretics. On imaging, tumours were identified in lungs, liver, spleen, kidneys, pancreas, adrenal glands and mediastinal lymph nodes. Lymph node biopsy revealed SCLC. Despite administration of three antihypertensive drugs, the patient’s blood pressure remained above 140/90 mmHg, potassium supplementation was started due to hypokalaemia and insulin due to hyperglycaemia. Palliative cytostatic treatment was started. Three days later the patient died with neutropenic sepsis despite treatment with broad spectrum antibiotics.

### Possible ectopic Cushing’s syndrome

Twenty-three patients (17 women) were considered to possibly have ECS; 12 patients with severe hypokalaemia, 9 patients with therapy resistant hypertension and two patients with new onset diabetes mellitus. The mean age at diagnosis in patients with possible ECS was 71 ± 8 years. Neither hyperpigmentation nor cushingoid features were documented in any of these patients. S-cortisol was measured in two, both having high concentrations at 8 AM (1,000 and 1,120 nmol/L, respectively).

### Survival

At the end of the study, 197 of 213 patients with SCLC had deceased. The median survival time was 13 months (range 1 day to 7.9 years).

**FIGURE 2:**
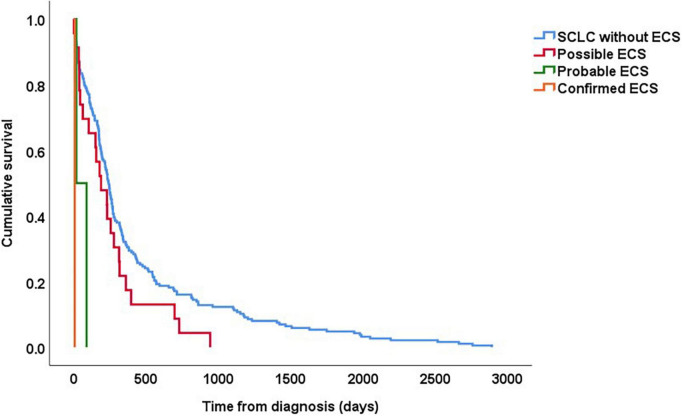
Kaplan–Meier curves showing survival in patients with SCLC based on whether they had “confirmed,” “probable,” and “possible” ECS. For all patients, follow-up started on the date of histological diagnosis.

The patient with confirmed ECS died 7 days after diagnosis, and the patients with probable ECS died after 19 and 89 days, respectively. The median survival time in patients with possible ECS was 8 months (range 1 day to 2.6 years), compared to 14 months (range 2 days to 7.9 years) in patients without suspicion of ECS (Figure 2).

## Discussion

In this retrospective study on an unselected cohort of 213 consecutive patients with SCLC we found a low prevalence of ECS; one patient was considered to have confirmed ECS, and two patients probably had the disorder. However, we also found that approximately 10% of the patients had clinical characteristics that are associated with ECS, i.e., either severe hypokalaemia, therapy resistant hypertension or new onset diabetes mellitus. We find it therefore likely that a substantial number of patients with SCLC may have had an undiagnosed ECS.

The prevalence of ECS among patients with SCLC has previously been studied in three relatively large studies (Table 3). In two of these studies, conducted at two different hospitals in Toronto Canada during the 1990’s, 23 of 545 (4.5%) and 14 of 840 (1.6%) patients with SCLC had ECS. More recently, 23 of 383 (6%) patients with SCLC from France were considered to have had ECS. Thus, between 1.6 and 6% of patients with SCLC develop ECS, where different definitions of ECS, as well as different thresholds for investigating the patients at individual centres, may explain some of the varying prevalence. However, given that all of these studies are retrospective, it is also likely that several cases of ECS remained undiagnosed. In our cohort only three of 213 (1.4%) patients with SCLC had either confirmed or probable ECS, also indicating an underdiagnosis of the disorder.

**TABLE 3:** Summary of previous studies describing the prevalence of ECS among patients with SCLC.

Author country (references)	Period	No of patients with SCLC	No (%) of patients with ECS	Diagnostic criteria for ECS	Median survival from diagnosis	Limitations
Nagy-Mignotte et al. (10) France	1998–2012	383	23 (6)	Two or more of the following: • P-cortisol >550 nmol/L • S-potassium <3.2 mmol/L • P-glucose >5.8 mmol/L (without prior history of diabetes) • P-ACTH >15 pmol/L • 24-h urinary free cortisol >300 nmol/day	6.6 months	Retrospective study
Delisle et al. (9) Canada	1971–1991	840	14 (1.6)	Two or more of the following: • P-cortisol >600 nmol/L, and loss of diurnal variation and/or lack of suppressibility by dexamethasone • S-potassium ≤3,2 mmol/L • P-ACTH >22 pmol/L • 24-h urinary free cortisol >400 nmol/day	5.5 months	Retrospective study
Shepherd et al. (8) Canada	1980–1990	545	23 (4.5)	Signs and symptoms of hypercortisolism and two or more of the following: • P-cortisol >660 nmol/L, and loss of diurnal variation and lack of suppressibility by dexamethasone • S-potassium <3 mmol/L • P-ACTH >22 pmol/L • 22-h urinary free cortisol >400 nmol/day	6.2 months	Retrospective study
Dimopoulos et al. (7) United States	1979–1989	90	11 (12)	Clinical and laboratory findings associated with hypercortisolism i.e., • Elevated corticosteroids • Hypokalaemia • Metabolic alkalosis • Muscle weakness • Diabetes • Hypertension	12 days from initiation of CHT	Study population limited to patients with SCLC who died within 90 days after chemotherapy was started; Definition of ECS was not formulated clearly

As far as we know, this is the first study aimed at estimating how often ECS may be undiagnosed in patients with SCLC. By collecting data on signs, symptoms and biochemical parameters that are characteristic for ECS, we identified 23 patients that possibly had undiagnosed ECS. Obviously, we cannot claim that all these patients had undiagnosed ECS. However, we would like to suggest that patients with SCLC, as well as patients with other neoplastic diseases associated with ECS, who present with severe hypokalaemia, therapy-resistant hypertension and/or new onset diabetes mellitus, should undergo biochemical testing to either confirm or exclude endogenous hypercortisolism. The hypercortisolism in patients with ECS is often severe where all the characteristic clinical features are present. This is, however, not the case in all patients, who instead present only with some of the features (7–10). In our opinion, the biochemical testing should therefore not only be performed in patients with pronounced hypercortisolism, but in all patients who present with either severe hypokalaemia, therapy resistant hypertension or new onset diabetes mellitus.

Small-cell lung cancer is a highly malignant disease with a poor prognosis and low 5-years survival rate (12). Among factors that have a negative influence on survival is the presence of ECS (7, 9, 10). In a study on survival in patients with SCLC, patients with ECS (*n* = 23) had a median survival of 6.6 months, which was significantly shorter compared with patients without any paraneoplastic syndrome (13 months) as well as patients with syndrome of inappropriate antidiuretic hormone secretion (8.5 months) (9). Also, in a retrospective study by Osswald, patients with ECS due to SCLC had a median survival rate of 5 months, which was significantly shorter than in patients with other forms of ECS (53–119 months) (13). In our cohort, the three patients with confirmed or probable ECS died within 3 months. In addition, the median survival time in patient with “possible” ECS was significantly shorter than in patients without a suspicion of ECS (8 v .14 months). We therefore consider it likely that some of these patients may have had undiagnosed ECS, although some of these patients may also have had a more advanced SCLC where e.g., hypokalaemia is more common than in patients in better general condition.

It is well known that patients with CS, irrespective of aetiology, have increased mortality (14–17). In many cases the cause of death is a direct consequence of the hypercortisolism *per se*, i.e., pulmonary embolism or sepsis, and not the underlying tumour (14). Thus, prompt and adequate treatment with cortisol-lowering drugs, thromboprophylaxis, prophylactic broad-spectrum antibiotics as well as prophylactic treatment against opportunistic microorganisms may improve the prognosis in many patients with ECS, and in some cases be life-saving. In fact, any diagnostic delay can be fatal. Furthermore, quality of life is severely impaired in patients with CS, and improves substantially following correction of the hypercortisolism (18). Therefore, it is essential to detect EAS as early as possible, initiate appropriate treatment, even in patient with incurable disease.

The major limitation of this study, as in all previous studies investigating the prevalence of ECS in patients with SCLC, is the retrospective design. Indeed, we cannot confirm that ECS is an underdiagnosed disorder, this is only an assumption. To solve the question a prospective trial, where consecutive patients with SCLC are screened for endogenous hypercortisolism, is needed.

## Conclusion

Our findings indicate that ECS is an underdiagnosed disorder in patients with SCLC. Given the serious consequences and poor prognosis of untreated ECS, the low detection rate highlights the need to improve knowledge of ECS among healthcare providers and to optimise early detection strategies for ECS.

## Data availability statement

The raw data supporting the conclusions of this article will be made available by the authors, without undue reservation.

## Author contributions

MP: data curation, formal analysis, investigation, methodology, project administration, software, visualisation, and writing – original draft preparation. ML, EP, and LO: writing – reviewing and editing. OR: conceptualisation, formal analysis, funding acquisition, methodology, resources, software, supervision, validation, visualisation, and writing – reviewing and editing. All authors contributed to the article and approved the submitted version.
